# Triggering Avoidance: Dissociable Influences of Aversive Pavlovian Conditioned Stimuli on Human Instrumental Behavior

**DOI:** 10.3389/fnbeh.2017.00063

**Published:** 2017-04-12

**Authors:** Sara Garofalo, Trevor W. Robbins

**Affiliations:** ^1^Behavioral and Clinical Neuroscience Institute, University of CambridgeCambridge, UK; ^2^Department of Psychiatry, University of CambridgeCambridge, UK; ^3^Department of Psychology, University of CambridgeCambridge, UK

**Keywords:** human Pavlovian-to-instrumental transfer, reinforcement learning, habits, goal-directed behavior

## Abstract

The present study investigates human aversive Pavlovian-to-Instrumental Transfer (PIT) and possible influences of outcome devaluation and instrumental overtraining on this effect. PIT measures the extent to which a Pavlovian conditioned stimulus (CS) can increase instrumental responses independently paired with the same (outcome-specific transfer) or a different (general transfer) reinforcer. Two measures of PIT were obtained: the percentage of instrumental responses and the vigor of such responses. Thirty-eight volunteers performed a standard PIT task sequence. Results showed a double dissociation between outcome-specific and general transfer: the first selectively expressed in the amount of responses, the second in the vigor measure solely. Furthermore, outcome-specific transfer was enhanced by overtraining, but not affected by devaluation. General transfer, on the other hand, was affected by neither overtraining, nor devaluation. A positive correlation between general transfer and sensitivity to punishments was found. Findings are discussed in terms of hypothetically different underlying neurobehavioral mechanisms and their relations to habits and goal-directed behavior.

## Introduction

Daily choices are influenced by environmental stimuli that signal the presence of potential punishments and rewards. The so-called Pavlovian-to-Instrumental Transfer (PIT) effect reflects the ability of a Pavlovian conditioned stimulus (CS)—i.e., a cue paired with a reinforcer—to increase the likelihood of an instrumental response independently paired with the same, or a similar, reinforcer (Rescorla and Solomon, 1967; Holmes et al., 2010). Thus, PT effect reflects the motivation acquired from a Pavlovian stimulus.

During Pavlovian conditioning, a CS is connected to the reinforcer both by a direct motivational representation of its value and an indirect representation of its sensory features (Dickinson and Balleine, 2002). This differentiation is thought to be reflected in two kinds of transfer effects: in a *general* form of transfer, the CS invigorates instrumental responses paired with motivationally similar reinforcers; whereas, in an *outcome-specific* form transfer, the CS exerts its influence selectively on instrumental responses associated with the exact same reinforcer.

There is a general lack of studies on PIT in human participants, especially in aversive contexts, so the present study aims to investigate the ability of aversive Pavlovian stimuli to increase the number and vigor of instrumental responses independently paired with the same (outcome-specific transfer) and different (general transfer) punishments.

The importance of a deeper understanding of the interactions between Pavlovian and instrumental learning processes comes from evidence suggesting that PIT effect may contribute to maladaptive behaviors, such as addiction (Hogarth et al., 2010, 2013b; Watson et al., 2013, 2014), compulsive behavior (Everitt and Robbins, 2005) and other neuropsychiatric disorders, like depression (Boureau and Dayan, 2011). From a clinical perspective, it appears important to understand how outcome-specific and general transfer affect goal-directed or habit-like behavior. Habits are indeed believed to be at the core of maladaptive behaviors characterized by a loss of control, such as addiction and compulsion (Everitt and Robbins, 2005).

A widely used procedure in literature for directly testing the goal-directed nature of an instrumental response is outcome devaluation (Balleine and Dickinson, 1998; Dolan and Dayan, 2013). Devaluation procedures weaken the value of a reinforcer, thus reducing goal-directed performance. Moreover, another variable that can favor the formation of habits is extended practice of the behavior, which can be experimentally reproduced by instrumental overtraining (Voon et al., 2014). Instrumental overtraining reduces the impact of the reinforcer on the instrumental responses, so that the performance is no longer guided by the current value of the outcome of the action (goal-directed), but is instead habitual. To further investigate the mechanisms behind transfer, a further aim of this study is to test the effect of outcome devaluation and instrumental overtraining on PIT.

Some authors have suggested that model-based/goal-directed decision making characterizes outcome-specific transfer, while model-free/habitual decision making characterizes general transfer (Dolan and Dayan, 2013). To our knowledge, this hypothesis has never been directly tested. If true, by promoting habitual behavior, devaluation and overtraining manipulations should decrease outcome-specific and, possibly, increase general transfer. However, it may could be argued that since both transfer effects implicate an external (Pavlovian) control over instrumental responses, they can hardly reflect a goal-directed behavior. In fact, transfer is observed in extinction, that is, in the absence of the goal itself. According to this alternative view, devaluation and overtraining could potentially have either no effect at all on outcome-specific and general transfer or increase both by boosting an already model-free/habitual decision-making process.

## Materials and Methods

### Participants

Thirty-eight volunteers (18 female; 4 left-handed; mean age = 25.18, sd = 5.69 years; mean education = 16.5, sd = 2.42 years) with no history of neurological diseases were recruited from the student population of the University of Cambridge (UK). The number of participants was determined based on *a priori* power analysis performed with G*Power 3.1 (Faul et al., 2009). The effect-size estimation was based on a previous study which investigated the same effect with a similar paradigm (Garofalo and di Pellegrino, 2015). All participants gave written informed consent to take part in the experiment and received payment corresponding to the amount of time needed to complete the tasks. The study was conducted in accordance with institutional guidelines and the 1964 Declaration of Helsinki and was approved by the Department of Psychology Ethics Committee of the University of Cambridge.

### Skin Conductance Response (SCR) Recording and Analysis

Ambu WS electrodes connected to a DC amplifier (Biopac Systems—MP150—GSR100) were used for recording galvanic skin response. These were attached to subjects’ volar surface of the index and middle fingertip in their left hand (which did not require any motor movement during the task). A gain factor of 5 μS/V and low-pass filter set at 10 Hz were used for recording the analog signal, which was then passed through the digital converter at a 200 Hz rate. The signal was then fed into AcqKnowledge 3.9 (Biopac Systems) and transformed into microsiemens for offline analysis. Skin Conductance Response (SCR) was extracted from the continuous signal and calculated for each trial as the peak-to-peak amplitude of the largest deflection during the 0.5–4.5 s time window following stimulus onset (Schiller et al., 2008). The minimal response criterion was 0.02 μS and smaller responses were encoded as zero. Raw SCR scores were square root transformed to normalize the distributions and scaled to each subject’s maximal response to the aversive stimulus, in order to account for inter-individual variability (Schiller et al., 2008).

Data were analyzed offline using custom-made MATLAB scripts (The MathWorks, Inc., Natick, MA, USA) and all statistical analyses were performed with RStudio v0.98.1062 (Boston, MA, USA). This signal was recorded to assess implicit Pavlovian learning. During the Pavlovian conditioning task all trials were recorded, however, analyses only included trials in which no aversive noise was delivered (40% of all CS+ and all CS− trials), in order to exclude artifacts.

### Hand-Grip and Response Recording and Analysis

An isometric hand dynamometer was used (Biopac Systems—MP150—TSD121C—DA100C) to record hand gripping (compression), by simply squeezing the handle of the transducer. To ensure correct recording of the hand-grip, the hand dynamometer was calibrated and each participant was familiarized with the maximum and minimum strengths that could be recorded. The hand-grip was recorded in kilograms and extracted from the continuous signal by calculating the maximum peak amplitude for each trial.

To allow for multiple responses, the hand dynamometer was attached to the base of a joystick and used as handle. In this way, participants could squeeze the handle-bar while moving it towards the left or the right. Both the side and the hand-grip were simultaneously recorded.

These measures were collected to obtain a measure of the vigor of all responses performed.

### Stimuli

**Figure 1:**
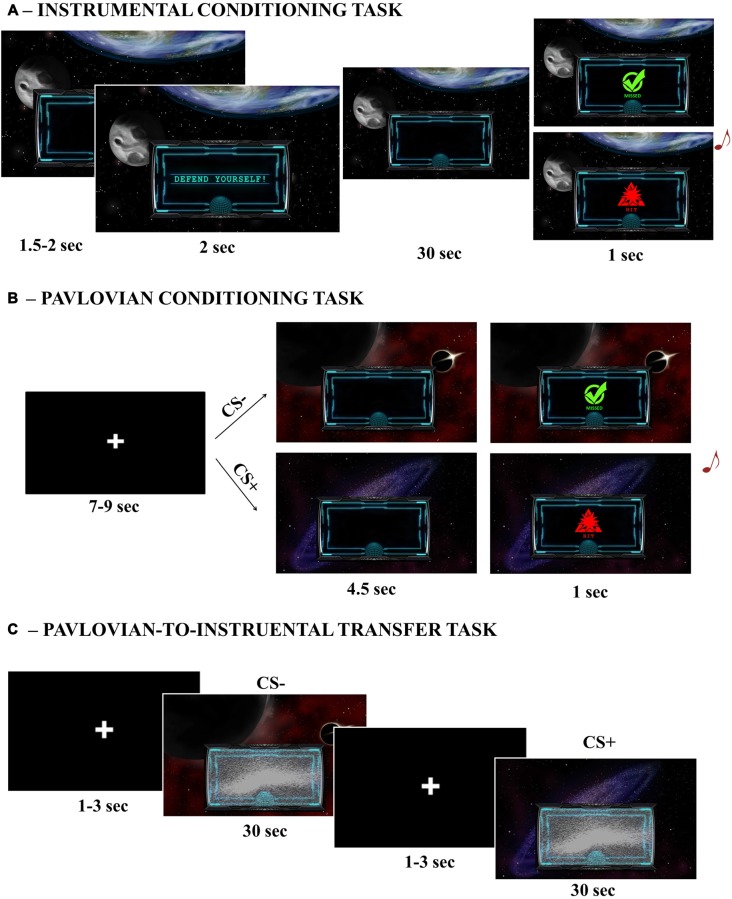
**Graphical illustration of the three main tasks.** Instrumental Conditioning task **(A)**, Pavlovian Conditioning task **(B)**; Pavlovian-to-Instrumental Transfer (PIT) task **(C)**.

Five different custom made images depicting space scenarios were presented in the background of a computer screen during all tasks (Figure 1). Centrally, in the lower part of the screen, a smaller image of a monitor was used to display visual feedbacks. Visual feedback consisted of: a green circle with the inscription “missed”; a red triangle with the inscription “hit”; the inscription “defend yourself”. Images were presented on a 17 inches computer screen, at a viewing distance of 80 cm.

The “hit” feedback was always paired with one of three different aversive noises, consisting in 100 db sounds played for 1 s. The three noises had been rated as equally aversive and clearly distinguishable by and independent group of subjects prior to the experiment.

A computer running Presentation software (Neurobehavioral Systems, Albany, CA, USA) controlled stimulus presentation.

### Procedure

On arrival, participants were comfortably seated in a silent room and their position was centered relative to the screen. They were required to wear a headset used to deliver aversive sounds during the task. Galvanic skin response, hand-grip force and behavioral responses were collected throughout the experiment and stored for offline analysis.

The experiment consisted of three main tasks. The tasks were presented in the following order: Instrumental Conditioning, Pavlovian Conditioning, PIT, PIT under devaluation (PIT-dev), Instrumental Overtraining, PIT after overtraining (PITo), PIT after overtraining under devaluation (PITo-dev). In each task, participants were required to pay attention to the screen and follow the instructions. A few example trials were always performed prior to each task. At the end of the experimental session, participants completed the Behavioral Inhibition/Activation System (BIS/BAS) inventory (Carver and White, 1994).

#### Instrumental Conditioning Task

Participants were engaged in a space-war game. In the initial instructions, participants warned that in this space mission they would be under attack and that their aim would be to find the right way to avoid such attacks. There were two possible sources of attack, which corresponded to two different aversive noises presented simultaneously with a “hit” visual feedback appearing in the small monitor for 1 s noises (Unconditioned Stimulus 1 and 2, or US1 and US2). The two USs were played prior the beginning of the task to allow familiarization. For the whole duration of the task, a single space scenario was presented on the background of the computer screen and, after a random inter-trial interval (1.5–2 s), a “defend yourself” message was prompted into the small monitor (2 s) to signal the beginning of a trial. For the following 30 s, only one of the two possible USs was randomly delivered according to a random time schedule (1.5–3 s). The US was consistent for the whole duration of the trial. To avoid attacks, participants were provided with a joystick and required to move it towards left or right, while squeezing. Their job was to figure out the correct response to avoid each specific US. Each side allowed avoidance of only one US (e.g., to avoid US1 the participant had to move the joystick to the left). The US could be avoided if the correct movement was performed at the time of US delivery. If the US was correctly avoided no noise was played and a “missed” visual feedback was displayed in the small monitor for 1 s (Figure 1A). The association between response (left/right) and attack (US1/US2) was counterbalanced across subjects. The rationale of this task was to learn the association between a specific US (US1/US2) and the correct response (left/right) required to avoid it. Participants performed four trials for each kind of attack, for a total of eight trials (of 30 s each) and a duration of about 5 min. At the end of this task, explicit learning was assessed by asking participants to pair each US with the corresponding correct avoidance response.

#### Pavlovian Conditioning Task

Participants were presented with new instructions informing that they would now be traveling through different galaxies (corresponding to the space scenarios used as CSs) and that more attacks could be delivered at this stage. They were also informed that they would not be able to use the joystick to avoid those attacks and were required to pay attention to the contingencies. In each trial, after a variable inter-trial interval (7–9 s), one of four possible space scenarios (CSs) was presented in background (4.5 s) and could be followed by either an aversive noise with a simultaneous “hit” visual feedback (1 s) or no noise with a simultaneous “miss” visual feedback (1 s). Two scenarios (CS + 1/CS + 2) were paired with the same two USs previously used during Instrumental Conditioning (US1 and US2); a third scenario (CS + 3) was paired with a new US (US3); a fourth scenario (CS−) was associated with no sound. All CS+ followed a 60–40 partial reinforcement schedule (Figure 1B). The association between CS and US was counterbalanced across subjects. The rationale of this task was to learn the association between the different space scenarios (CSs) and each US. Participants performed 20 trials for each CS condition, for a total of 80 trials and a duration of about 15 min. At the end of this task, explicit learning was assessed by asking participants to pair each US with the corresponding CS.

#### Pavlovian-to-Instrumental Transfer (PIT) Task

Participants were instructed that at this stage they could use again the joystick to avoid attacks (as during the instrumental conditioning task), but that a malfunction occurred to the small monitor and no visual feedback was going to be displayed. The task was identical to the Instrumental Conditioning task, except for two aspects: first, the task-irrelevant space scenarios used during Pavlovian Conditioning as CSs were randomly presented in background, one for each trial; second, the task was completely performed under extinction, so neither visual feedbacks nor aversive noises ever occurred (Figure 1C). The rationale of this phase is to test the ability of a task-irrelevant Pavlovian cue to trigger avoidance responses (presumably, towards the one previously associated with the same or a similar punishment) even if no aversive stimuli are ever delivered (i.e., extinction). Extinction is a standard procedure for assessing transfer, both in human and animal PIT research, since it allows one to test the influence of Pavlovian cues on instrumental responding without the confounding effects of the reinforcer (Bray et al., 2008). Participants performed four trials for each CS condition, for a total of 16 trials and a duration of about 8 min.

#### PIT with Devaluation

In this phase, the PIT task was repeated exactly as before, but the US was devalued by removing the headset prior the beginning of the task so that no USs could be delivered. A similar procedure for USs devaluation was successfully used in previous studies, where the subject is physically disconnected from the source of aversive stimulation in order to reduce its value (Gillan et al., 2014).

#### Instrumental Overtraining

During this task, participants tripled the training performed during Instrumental Conditioning task, for a total of 24 trials of 30 s each, during which multiple responses were performed (on average, 400 instrumental responses for the whole duration of this phase).

## Results

### Instrumental Conditioning

**Figure 2:**
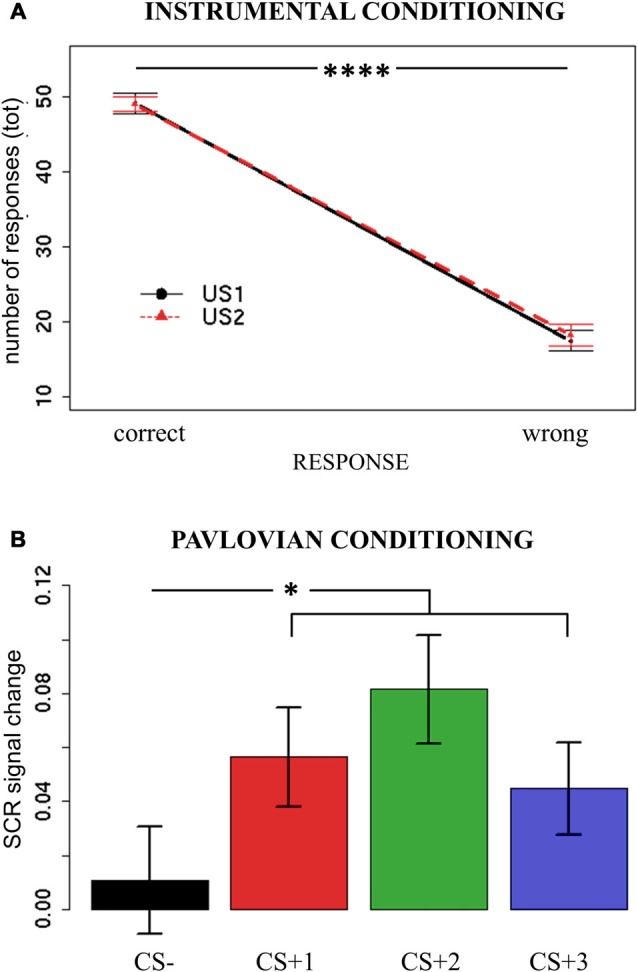
**Instrumental Conditioning and Pavlovian Conditioning results.** Panel **(A)** reports the number of correct and wrong responses performed when presented with the two different attacks/noises (Unconditioned Stimuli—USs) during Instrumental Conditioning. Panel **(B)** reports the Skin Conductance Response (SCR) signal change (second hemiblock—first hemiblock) when presented with all possible conditioned stimuli (CSs) during Pavlovian Conditioning. Bars indicate standard error of the mean. *p < 0.05, ****p < 0.001.

To assess implicit learning of the Instrumental Conditioning task, the numbers of responses performed with the joystick were analyzed. For each trial, a response (left/right) was categorized as correct or wrong according to its ability to avoid the current attack (US1/US2). Each side was allowed to avoid only one specific US, uniquely associated with a particular attack. A mixed-effects model was used, with Response (correct/wrong) and US (US1/US2) as independent variables, and the total number of responses as dependent variable. Subjects were modeled as a random effect. Assumptions of normal distribution, independence of residuals and sphericity were verified. Results showed a main effect of Response (*F*_(1,37)_ = 194.4; two-tailed *p* < 0.0001; part-*η*^2^ = 0.84), with more correct responses (mean = 49.17; sd = 7.41) being performed than wrong responses (mean = 17.82; sd = 8.49; Figure 2A). All other effects were not significant (*p*s > 0.58). Moreover, 89% of participants made the correct responses when explicitly asked to indicate the avoidance response associated with each US.

These results indicate that participants learned, both implicitly and explicitly, to discriminate between the two USs and the corresponding avoidance response.

Acquisition of the instrumental contingencies over time is reported in the supplementary materials.

### Pavlovian Conditioning

To assess implicit learning during the Pavlovian Conditioning task, an SCR signal change index was calculated, to obtain a measure of changes in arousal level as learning occurred. To detect variations in time, the difference between SCR during the second and first hemiblocks of the task was calculated (SCR signal change) for each CS. If participants correctly learned to discriminate between aversive and neutral Pavlovian cues, a higher signal change should be observed for all CS+ trials relative to CS− trials. A mixed-effects model was used, with CS (CS + 1/CS + 2/CS + 3/CS−) as independent variables, and SCR signal change as dependent variable. Subjects were modeled as random effect. Assumptions of normal distribution, independence of residuals and sphericity were verified. Results showed a significant main effect of CS (*F*_(1,111)_ = 3.17; two-tailed *p* = 0.03; part-*η*^2^ = 0.08). Bonferroni-corrected *post hoc* analysis revealed a significant difference between CS− (mean = 0.011; sd = 0.12) and all CS+ (CS + 1 mean = 0.056, sd = 0.11; CS + 2 mean = 0.081, sd = 0.12; CS + 1 mean = 0.044, sd = 0.10) conditions (respectively, *p* = 0.03; *p* = 0.01; *p* = 0.04), but not between the CS+ (*p*s > 0.2; Figure 2B).

These results show that, as learning occurred over time, participants’ arousal significantly increased when presented with aversive stimuli (all CS+) as compared to a neutral stimulus (CS−), thus indicating successful Pavlovian conditioning.

### Pavlovian-to-Instrumental Transfer

To assess the PIT transfer effect, two dependent variables were used, the percentage choice of responses and the hand-grip force.

**Figure 3:**
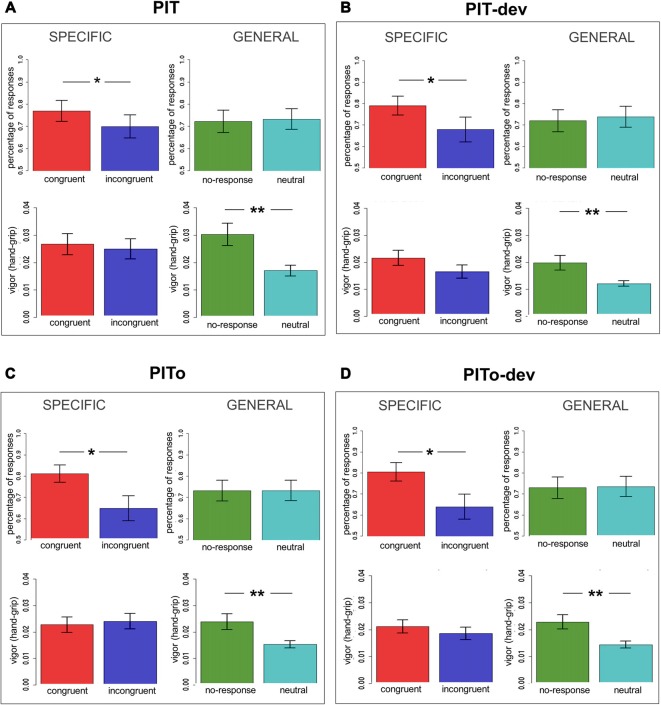
**PIT effect across tasks.** Panel **(A)** shows the first transfer task performed (PIT); panel **(B)** shows PIT under devaluation (PIT-dev); panel **(C)** shows PIT after overtraining (PITo); panel **(D)** shows PIT after overtraining under devaluation (PITo-dev). All panels show results for outcome-specific and general transfer on both percentage of responses and hand-grip. Bars indicate standard error of the mean. PIT, Pavlovian-to-Instrumental Transfer. *p < 0.05; **p < 0.01.

Specific transfer was tested considering only CS + 1 and CS + 2 trials, as these were paired with the same USs used during Instrumental Conditioning. The rationale of outcome-specific transfer is to test if CSs are able to elicit a response independently associated with *the same* reinforcer. For this aim, all responses were categorized as congruent (e.g., choosing R1 when presented with CS + 1 or choosing R2 when presented with CS + 2) or incongruent (e.g., choosing R2 when presented with CS + 1 or choosing R1 when presented with CS + 2) and compared. Two separate mixed-effects models were used, with Congruency (congruent/incongruent) as independent variable and percentage of responses or hand-grip as dependent variables. Subjects were modeled as a random effect. Assumptions of normal distribution, independence of residuals and sphericity were verified. Results (Figure 3A) for mean percentage of responses showed a significant difference (*F*_(1,37)_ = 2.56; two-tailed *p* = 0.05; part-*η*^2^ = 0.12) between congruent and incongruent responses, with the former being more numerous than the latter. Hand-grip force showed no difference (*p* = 0.7) between congruent and incongruent responses (means are reported in Figure 3A). In sum, outcome-specific transfer effect was observed when considering response frequency (percentage of responses), but not when considering the vigor (hand-grip) of such responses.

General transfer, on the other hand, was tested considering only CS + 3 and CS− trials, as these were respectively paired with the US not used during Instrumental Conditioning (no-response condition) and with no US (neutral condition). The rationale of general transfer is to test if CSs are able to elicit a response independently associated with *a similar* reinforcer (no-response), relative to a neutral CS. Two separate mixed-effects models were used, with CS (no-response/neutral) as independent variable and percentage of responses or hand-grip as dependent variables. Subjects were modeled as a random effect. Assumptions of normal distribution, independence of residuals and sphericity were verified. Results for mean percentage showed no difference (*p* = 0.5) between no-response and neutral responses (Figure 3A). Results for hand grip (Figure 3A) showed a significantly greater (*F*_(1,37)_ = 37.18; two-tailed *p* < 0.001; part-*η*^2^ = 0.51) force for no-response vs. neutral responses. In sum, the general PIT transfer effect was only observed when considering the vigor (hand-grip) of responses, but not when considering the frequency of such responses (percentage of responses). This result indicates a double dissociation between outcome-specific and general aversive transfer effects according to the index of measurement employed.

All the following transfer tasks (PIT-dev, PITo, PITo-dev) were analyzed following the same criteria and showed a similar trend. All results are described in detail in Table 1 and means are reported in Figures 3B–D.

**Table 1:** **Outcome-specific and general transfer results in all tasks**.

		Percentage of responses			Hand-grip		
		*F*_(1,37)_	*p*	part-*η*^2^	*F*_(1,37)_	*p*	part-*η*^2^
PIT	Specific	2.56	0.05	0.12	0.61	0.44	0.02
	General	0.2	0.6	0.01	26.19	<0.001	0.42
PIT-dev	Specific	5.67	0.02	0.13	0.13	0.72	0
	General	0.43	0.52	0.01	37.18	<0.001	0.51
PITo	Specific	14.06	<0.001	0.28	0.86	0.36	0.02
	General	0	0.9	0	45.03	<0.001	0.55
PITo-dev	Specific	13.85	<0.001	0.27	0.18	0.67	0.01
	General	0.04	0.83	0	65.87	<0.001	0.64

*PIT, Pavlovian-to-Instrumental Transfer; PIT-dev, PIT under devaluation; PITo, PIT after overtraining; PITo-dev, PIT after overtraining under devaluation*.

### Transfer Effect Across Tasks

**Figure 4:**
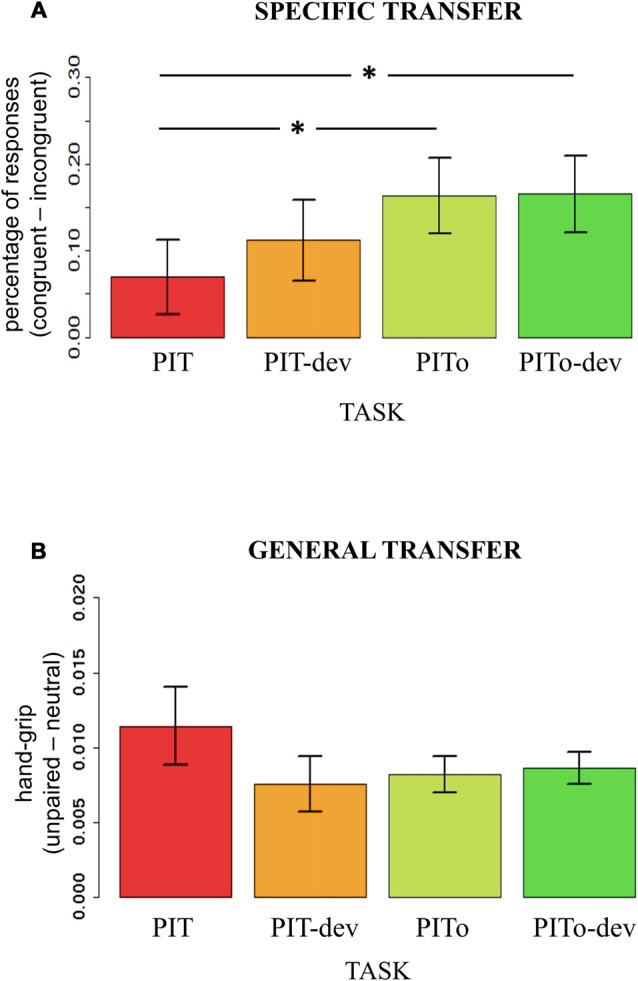
**PIT.** Panel **(A)** shows an index of the outcome-specific transfer effect calculated on percentage of responses (Congruent-Incongruent). Panel **(B)** shows an index of the general transfer effect calculated on hand-grip (Congruent-Incongruent). PIT, Pavlovian-to-Instrumental Transfer; PIT-dev, PIT under devaluation; PITo, PIT after overtraining; PITo-dev, PIT after overtraining under devaluation. Bars indicate standard error of the mean. *p < 0.05.

To test how transfer effects on both percentages of responses and hand-grip were modulated by the experimental manipulations of devaluation and instrumental overtraining, an index of transfer effect was computed. For outcome-specific transfer, the index was calculated on the percentage of responses (i.e., using the dependent variable that expressed outcome-specific transfer effect) by subtracting incongruent from congruent responses. For general transfer, the index was calculated for the hand-grip force (i.e., using the dependent variable that expressed an effect) by subtracting responses during neutral trials (neutral) from responses during aversive trials (no-response). Two separate mixed-effects models were used, with Task (PIT/PIT-dev/PITo/PITo-dev) as the independent variable and outcome-specific or general transfer index as dependent variables. Subjects were modeled as a random effect. Assumptions of normal distribution, independence of residuals and sphericity were verified. Results for outcome-specific transfer showed a significant main effect of Task (*F*_(2.3,85.19)_ = 3.53; two-tailed *p* = 0.03; part-*η*^2^ = 0.09; Figure 4A). Bonferroni-corrected *post hoc* analysis revealed a significant difference (*p* = 0.04) between PIT (mean = 0.07; sd = 0.26) and PITo (mean = 0.16; sd = 0.26) and between PIT and PITo-dev (mean = 0.17; sd = 0.27; *p* > 0.03). Results for the general transfer index reported no significant differences between the tasks (*p* = 0.2; Figure 4B).

These results indicate that outcome-specific transfer was enhanced by instrumental overtraining, but not when reinforcer devaluation had occurred, and, conversely, general transfer was affected neither by instrumental overtraining nor reinforcer devaluation.

### Sensitivity to Punishments and Rewards: Correlation with BIS/BAS Inventory

**Figure 5:**
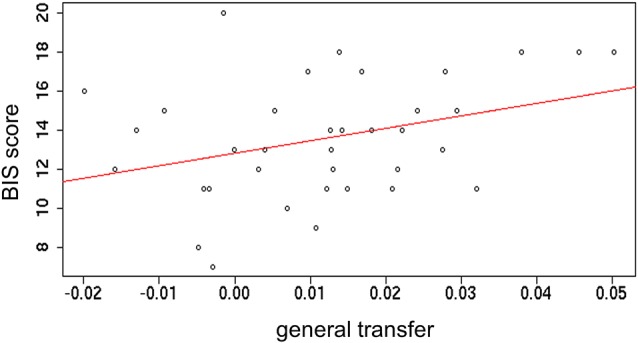
**Correlation between the index of general transfer effect and the Behavioral Inhibition System (BIS) scale**.

To further investigate the PIT transfer effect, correlations with sensitivity to punishments and rewards, as captured from the BIS/BAS inventory (Carver and White, 1994) were tested. Outcome-specific and general transfer indices (obtained during the first time transfer task was performed) were separately correlated with both BIS and BAS subscales. A small significant positive correlation between general transfer and BIS was found (*r* = 0.34; *p* = 0.04). All other correlations were not significant (*p*s > 0.1; Figure 5). This result indicates that stronger motivation to avoid potential punishments is linked to a stronger sensitivity to such punishments.

## Discussion

In the present study, a double dissociation between outcome-specific and general aversive PIT in human participants was found for the first time. Outcome-specific transfer was expressed by the relative proportion of responses (percentage), but not the vigor (force) measure; whereas the opposite pattern was observed for general transfer, i.e., expressed in the vigor, but not in the proportion of responses. Moreover, whereas outcome-specific transfer was enhanced by instrumental overtraining, but not by reinforcer devaluation, general transfer was affected by neither instrumental overtraining, nor reinforcer devaluation. A positive correlation between general transfer and sensitivity to punishments (as measured by the BIS scale) was also found. These findings will be discussed in terms of the hypothetically different underlying neurobehavioral mechanisms for general and outcome-specific PIT and how they interact with goal-directed behavior and habit based stimulus-response learning.

A previous human study (Watson et al., 2014) also measured outcome-specific and general transfer with percentage and vigor of responding, respectively, reporting clear evidence for the first (food-associated cues were able to bias choice toward the signaled food) and less clear results for the second (general transfer was weak and modulated by the individual motivational state). However, Watson et al. (2014) did not directly demonstrate a double dissociation between the two measures’ ability to capture outcome-specific and general forms of PIT. Critically, the vigor of responding was operationalized simply as the response rate, whereas in the present study a more direct measure of the force exerted for each response (hand-grip) was used.

To our knowledge, only a few studies have analyzed PIT in an aversive context before this one (Nadler et al., 2011; Trick et al., 2011; Lewis et al., 2013). In contrast with the present results, two studies (Nadler et al., 2011; Lewis et al., 2013) reported a general form of transfer when looking at response rate. However, general transfer was assessed by measuring rate of responding over instrumental baseline levels, whereas in the present study the number of responses was simply compared across conditions and not referred to any baseline. A concealing interpretation may be that calculating the response increase over baseline levels, to some extent, resembles more a form vigor rather than a simple calculation of the number of responses. In this view, these results are not in total contrast with the present results, although it would still remain unclear why the same “vigor” measure also highlights outcome-specific transfer. Overall, the presence of several methodological differences makes it difficult to directly compare results across these studies. Just to name some: in Nadler et al. (2011) a quasi-avoidance procedure was used (outcomes were unsignaled) with primary reinforcers (shocks); Lewis et al. (2013) used a complete avoidance procedure, but secondary reinforcers (instructed) were used; Trick et al. (2011) measured the transfer effect by comparing the number of avoidance responses, but the procedure used makes it impossible to disentangle between specific and general PIT.

Evidences from human neuroimaging studies support the conclusion of a dissociation between outcome-specific and general PIT, as separate and functionally coherent neural substrates, have been associated with these two forms of transfer. More specifically: activity in the dorsal striatum and ventral amygdala correlates with outcome-specific PIT (Bray et al., 2008; Prévost et al., 2012); whereas, activity in the ventral striatum and dorsal amygdala correlates with general PIT (Talmi et al., 2008; Prévost et al., 2012). Overlapping results were also specifically reported for the aversive form of PIT (Lewis et al., 2013). However, results from non-human studies, mainly focusing on appetitive PIT, are not so clear-cut. Within the striatum, dorsal (Corbit and Janak, 2007) and ventral (Corbit et al., 2001; Hall et al., 2001; Corbit and Balleine, 2011) sectors have been inconsistently associated with outcome-specific and general forms of PIT. Within the amygdala, conversely, a clearer differentiation has been observed, with the basolateral amygdala being selectively involved in outcome-specific PIT (Hall et al., 2001; Holland and Gallagher, 2003; Corbit and Balleine, 2005) and the central nucleus selectively involved in general PIT (Hall et al., 2001; Holland and Gallagher, 2003; Corbit and Balleine, 2005).

Overall, it is remarkable that overlapping regions in the mediation of PIT effects have been identified across species from humans to rodents, which suggest functional conservation across species and support the potential value of translational studies. However, more studies are needed to clarify such relationships and the underlying mechanisms.

### Effects of Devaluation

In accordance with the hypothesis that transfer does not reflect intentional goal-directed behavior, the present experiment reported no effect of aversive US devaluation on either outcome-specific or general transfer in humans, regardless of the amount of training (thus, both before and after instrumental overtraining).

Previous studies obtaining similar results with animals reported a variety of possible interpretations. For some of these authors, devaluation is postulated to act on the sensory specific properties of the reinforcer, rather than reducing any motivational influence that a Pavlovian CS (associated with a reinforcer before its devaluation) exerts on instrumental responding during PIT (Holland, 1990, 2004; Colwill and Motzkin, 1994). Conversely, other authors have preferred the alternative view that decreases in motivational value of the US consequent to devaluation procedure should not affect PIT, as the role of the mediating outcome is seen as a mere step in a chain of events that activates the response (stimulus → outcome representation → response representation; Rescorla, 1994).

The controversy may be resolved by taking into account details of the different devaluation procedures used. Previous studies investigating the impact of devaluation on transfer effect in humans reported contrasting results. Watson et al. (2014) reported that food satiation did not influence either outcome-specific or general PIT effects. Allman et al. (2010), on the other hand, found a reduced outcome-specific transfer for stimuli associated with a devalued currency. Another recent experiment (Eder and Dignath, 2016) observed that outcome-selective transfer was reduced by outcome devaluation (taste aversion) only when the devalued outcome was consumed immediately after each test phase, but not when its consumption was delayed.

Such different results may be explained by the use of very different devaluation procedures. Devaluation procedures using satiety might act more on the general motivation towards food (satiation for popcorn might reduce not only craving for popcorns but also general hunger). Whereas, a procedure like currency deflation might act more selectively on the specifically devalued currency, rather than on the general motivation towards monetary gains (devaluing a currency does not impair the desire to win more money, especially if there is a more valuable currency available). These different procedures do complicate the interpretation of the results.

In the present experiment devaluation (headset removal) led to the impossibility for the learned aversive reinforcers to be delivered, which should impact both general fear (motivation) and aversion for those precise (sensory-specific) aversive stimuli. In the present experiment, it is not possible to disentangle between these two possibilities, but future studies might directly address this interesting issue. However, despite acting on both motivation and sensory-specific features, the devaluation procedure used in the present study failed to affect transfer. Consequently, it may be argued that actions which are not altered by such a manipulation resemble a more “S-R habit-like” behavior, being not driven by the value of the potential reinforcer.

Overall, the absence of an effect of devaluation on both outcome-specific and general transfer reported here reflects the independence of transfer from the current value of a reinforcer, hence adding to the conclusion that the influence exerted by Pavlovian CSs on instrumental responses does is not goal-directed (Hogarth et al., 2013a).

### Effects of Overtraining

A possible different mechanism underlying outcome-specific and general transfer are also reflected (other than by the double dissociation discussed above) by the diverse sensitivity of the two transfer forms to instrumental overtraining. Overtraining increased outcome-specific transfer but did not alter general transfer.

Instrumental overtraining is believed to favor the formation of habits at the expenses of goal-directed behavior (Dickinson et al., 1995; Yin and Knowlton, 2006; Tricomi et al., 2009; Dayan and Berridge, 2014). Overtraining is, indeed, interpreted as a progressive shift in control of instrumental responding from a direct R-O association to a more indirect S-R associations, which overcomes and weakens the reinforcer representation (Holland, 2004). In line with this interpretation, overtraining here increased outcome-specific transfer, driven by an S-O-R association (thus, mediated by the outcome representation), but did not alter the general transfer effect, driven by an S-R association (for which no representation of the outcome is involved). As a result, in the present study, instrumental overtraining weakened the outcome representation involved in the outcome-specific transfer causing a boost of the habitual effect but had no impact on general transfer (where the outcome representation is irrelevant).

Another possibility is that the extra amount of training that strengthen the R-O association, thus increasing a goal-directed outcome-specific transfer. However, this interpretation seems unlikely given the vast literature about the effects of overtraining (Dickinson et al., 1995; Yin and Knowlton, 2006; Tricomi et al., 2009; Dayan and Berridge, 2014) and the lack of difference in accuracy between instrumental conditioning and instrumental overtraining in the present task (see, Supplementary Materials, Figure S1).

Conclusions about the effects of devaluation and overtraining should, however, be made cautiously. A limitation of this study is, indeed, represented by the lack of direct validation of both devaluation and overtraining procedures. The operationalization of devaluation and overtraining mirrored those described and successfully implemented in previous studies (see “Procedure” Section), but the absence of a direct measure of their effectiveness in this experiment still represents a possible source of error that must be taken into account when discussing these results.

### Sensitivity to Punishments

A positive correlation was found here between general transfer and the BIS inventory, which measures sensitivity to signals of punishment (Carver and White, 1994).

The underlying principle of the BIS/BAS inventory is that behavior is guided by two separate regulatory systems: the approach system (captured by the BAS) and the withdrawal system (captured by the BIS; Carver et al., 2000). The first one processes positive affect and goal pursuit; the second one handles negative affect and avoidance of threat. These two systems can prompt actions and underpin a tendency to chase goals or to avoid threats, respectively (Carver et al., 2000). The finding of a correlation between general transfer and BIS score indicates that individual differences in the responsivity to punishments can inform about the extent to which individual choices are influenced by external cues that acquired the same motivational value (i.e., aversive cues in the present experiment).

### Conclusion

The presence of a double dissociation between outcome-specific and general forms of transfer selectively expressed in the quantity (percentage) and the vigor measure (force), respectively, is consistent with the existence of different underlying learning mechanisms for outcome-specific and general transfer.

The evidence that devaluation failed to affect outcome-specific and general forms of transfer is consistent with the hypothesis that both forms of transfer may not reflect a goal-directed choice, but rather habitual behavior, as the bias induced by the Pavlovian cue was not linked to the current value of the outcome. The increase in outcome-specific transfer—though not general transfer—after instrumental overtraining points, on the one hand, to a habit account of PIT, while on the other hand supports the idea that dissociable aspects of the associative representations underlie the two forms of PIT: outcome-specific transfer is mediated by an outcome representation that can be weakened by overtraining, while no outcome representation is involved in general transfer. However, the limitations discussed above concerning these experimental manipulations should be carefully taken into account.

In conclusion, cautious consideration should precede definitive interpretation of a complex phenomenon such as the PIT effect. More studies are needed to clarify the differences arising from the many devaluation procedures used in the literature (Eder and Dignath, 2016) and the disparity of methods used in the different PIT studies speaks for the necessity of cautious conclusions and the need for more standardized procedures and replication studies.

## Author Contributions

SG and TWR conceived and designed the experiment; read, modified, corrected and approved the final manuscript; SG programmed the task, ran the experiment, analyzed the data, wrote the main manuscript text and prepared the figures.

## Conflict of Interest Statement

The authors declare that the research was conducted in the absence of any commercial or financial relationships that could be construed as a potential conflict of interest.
