# Supplementary material for: Triggering Avoidance: Dissociable Influences of Aversive Pavlovian Conditioned Stimuli on Human Instrumental Behavior
Source: Front Behav Neurosci. 2017 Apr 12;11:63. doi: 10.3389/fnbeh.2017.00063 (PMC5388761; doi:10.3389/fnbeh.2017.00063)
Supplement: Supplementary file 1 [file DataSheet_1.doc]

##### SUPPLEMENTARY MATERIAL

Performance during Instrumental Conditioning and Overtraining was also evaluated across time, by dividing, within each task, the whole duration into 10 equal time-bins (Fig. S1). For each task, a mixed-effects model was used, with Response (correct/wrong), Unconditioned Stimulus (US1/US2) and Time-bin (1-10) as independent variables; and the total number of responses as dependent variable. Subjects were modeled as a random effect. Assumptions of normal distribution, independence of residuals and sphericity were verified. Results on the Instrumental Conditioning task showed a main effect of Response (F(1, 37) = 56.7; two-tailed p < .0001; part. η2 = .9), with more correct responses (mean =9.83; sd =2.41) being performed than wrong responses (mean =5.56; sd =2.64) (Fig. S1-A). All other effects were not significant (ps > .05). Results on the Instrumental Overtraining task showed a main effect of Response (F(1, 37) = 197.9; two-tailed p < .0001; part. η2 = .9), with more correct responses (mean =9.07; sd =2.19) being performed than wrong responses (mean =2.76; sd =2.02) (Fig. S1-B). All other effects were not significant (ps > .05).

Moreover, the percentage of correct responses (calculated as the number of correct responses over the total number of responses) was compared between the two tasks. A mixed-effects model was used, with Task (Instrumental Conditioning/Overtraining) and Time-bin (1-10) as independent variables; and the percentage of correct responses as dependent variable. Subjects were modeled as a random effect. Assumptions of normal distribution, independence of residuals and sphericity were verified. Results showed a significant interaction between Task and Time-bin (F(5.87, 217.15) = 7.5; two-tailed p < .0001; part. η2 = .17) (Fig. S1-C). Bonferroni-corrected post hoc analysis reported a significant difference between Instrumental Conditioning and Overtraining within the first and the second time bin (ps < .0001).

#####
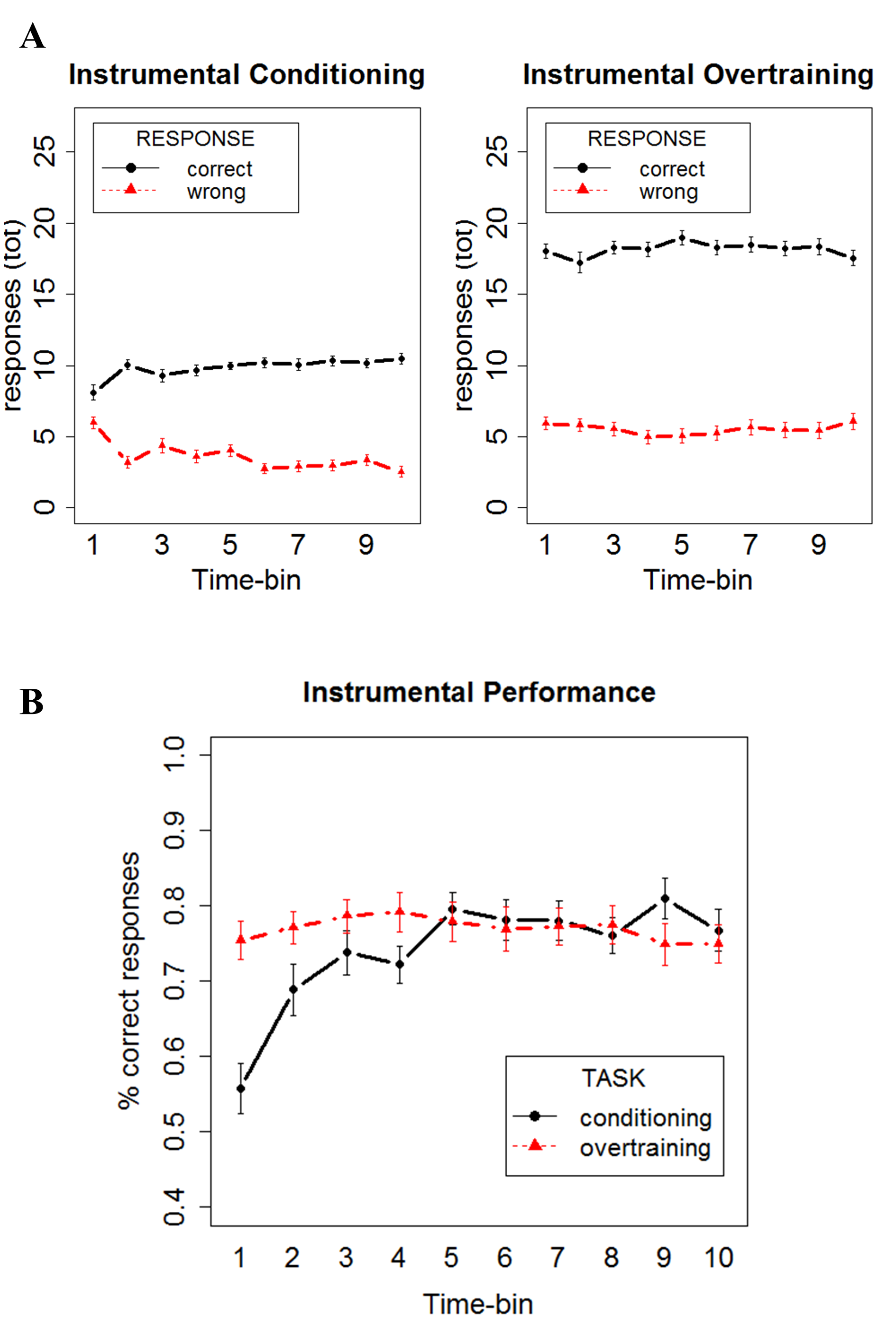


##### Figure S1. Performance during Instrumental Conditioning and Overtraining across time (10 time-bins). Bars indicate standard error of the mean.

##### Panel (A) shows the total number of correct and wrong responses over time

##### during Instrumental Conditioning and Overtraining independently.

##### Panel (B) shows the percentage of correct responses over time,

##### compared between Instrumental Conditioning and Overtraining tasks.
